# The Lebanese Society of Medical Oncology (LSMO) statement on the care of patients with cancer during the COVID-19 pandemic

**DOI:** 10.2217/fon-2020-0252

**Published:** 2020-04-08

**Authors:** Nizar Bitar, Joseph Kattan, Hampig Raphael Kourie, Deborah Mukherji, Nagi El Saghir

**Affiliations:** 1 Lebanese Society of Medical Oncology (LSMO), Beirut, Lebanon; 2 Hematology-Oncology Department, Hôtel Dieu de France University Hospital, Faculty of Medicine, Saint Joseph University of Beirut, Lebanon; 3 Department of Internal Medicine, Division of Hematology-Oncology, American University of Beirut Medical Center (AUBMC), Beirut, Lebanon

**Keywords:** cancer patients, COVID-19, lebanon, oncology staff, recommendations

COVID-19 is a highly transmissible viral illness caused by SARS-CoV-2 of the coronavirus family. This disease is highly transmissible mostly through coughing and sneezing droplets, contaminated surfaces and aerosols. Airborne transmission remains unclear however the virus may survive in the droplets in the air for a short time [1].

Patients with a diagnosis of cancer on active antineoplastic therapy are particularly vulnerable to viral infections, particularly the immunosuppressed. Patients who require assistance with activities of daily living may also be more vulnerable to contracting viral infections from close contacts. The risk of hospitalization is four-times higher, and the risk of death is ten-times higher compared with the general population, particularly in patients with hematologic malignancy or those receiving many lines of chemotherapy or presenting neutropenia and lymphopenia [2].

According to recently published data from China from a small cohort of patients, there was no increase in incidence of COVID-19 infection in cancer patients but cancer patients had a higher incidence of severe events; the risk of hospitalization for cancer patients were higher compared with the general population (1% vs 0.29%). Moreover, in infected patients with COVID-19, their risk of developing respiratory complications necessitating intensive care was higher in cancer patients compared with noncancer patients (39% vs 8% ; p = 0.003) [3]. A recent report from Italy has shown that almost 20% of over 3000 patients who died after contracting COVID-19 had a history of active cancer within the past 5 years [4].

Based on these results, it is important to establish urgent local guidelines and recommendations for caring for cancer patients during the COVID-19 outbreak period based on their disease stage, site and its specific treatment.

In view of the evolving spread of COVID-19 in Lebanon resulting in a ‘State of Emergency’ declared by the government and health authorities, the Lebanese Society of Medical Oncology (LSMO) has issued rapid guidance notes for management of patients with cancer. These have been based on published experience from China, Italy and other countries affected by COVID-19. LSMO would like to share these suggested guidelines and list of resources with all healthcare practitioners, particularly in limited resource settings where mass testing of asymptomatic patients and staff is unavailable.

Our pragmatic recommendations for daily practice for the care of cancer patients during COVID-19 pandemic relate to prevention of contamination, prioritization of patients, avoiding overcrowded clinics, ensuring the separation of oncology departments from other units and management of palliative care patients.

1. Prevention of contamination (patients and staff): Screening of patients and visitors for travel/contact history and symptoms. DO NOT ADMIT COVID-19 positive patients and suspected cases to the outpatient department or the oncology floor. Refer suspected and infected cases to COVID-19 specialized departments and services for management.
2. Prioritization of patients by favoring curative therapies versus palliative, application of therapy pause when justified and limiting chemotherapy and immunotherapy for patients with poor prognosis. We are recommending discussions with all patients with advanced disease regarding additional risks versus benefits of further lines of therapy.
3. Avoid overcrowded clinics (by deferring regular routine follow-up to over-the phone consultations) and chemotherapy units by decreasing the number of patients receiving weekly chemotherapy versus more spaced regimens where possible, consider switch to oral chemotherapy to be taken at home when possible versus intravenous treatment.
4. Separation or ‘sanctuarization’ of the oncology department: withhold any immunosuppressive treatment of patients diagnosed to have COVID-19/high risk for development of disease until full recovery. Admission of COVID-19 positive should be done in specialized departments.
5. Manage patients in need of supportive care and palliation through phone calls to keep them safe at home.

We suggest different strategies in various settings to take care of cancer patients based on their disease stage and the treatment they have received (curative vs palliative) [5]. The detailed suggested strategy for patients on follow-up or on endocrine/nonimmunopressive oral targeted therapies who can be managed at home, patients with early stage cancer/curative setting and patients with metastatic disease are summarized in the Table 1.

**Table 1.** Suggested strategies in different settings in cancer patients during COVID-19 pandemic.

Setting	Suggested Strategy
Patients on follow-up or on endocrine/oral targeted therapies	• Prevention • Delay visits and follow-up appointments in absence of active disease/new symptoms requiring review • Delay routine re-staging imaging if no new symptoms • Lab tests can be performed locally if required and reviewed by telephone/sending picture of results • Telephone contact/telemedicine in place of clinic visits
Patients with early-stage cancer/curative setting	• Prevention • Close monitoring for potential toxicity and for COVID-19 clinical symptoms • Consider increased use of GCSF to limit neutropenia • Discussion of risks versus benefits of adjuvant therapies with patients • Consider limiting duration of adjuvant therapy where appropriate (3 vs 6 months adjuvant chemotherapy for ‘good risk’ stage 3 colon cancer for example) • Choose three weekly regimens instead of weekly regimen
Patients with metastatic disease	• Prevention • Close monitoring for potential toxicity and for COVID-19 clinical symptoms • Consider delay in treatment or therapeutic break if not compromising disease control • Consider oral therapy options and telemedicine for toxicity management • Discuss risks versus benefits with patients

The prevention advice for patients consist of avoiding crowded places, washing hands according to WHO recommendations, using sanitizers and gloves, wearing masks properly when going to clinic/hospital. Moreover, cancer patients should not have any contact with family and friends with COVID-19 symptoms/possible exposure and should practice social distancing with all people to protect themselves and others. Finally, patients should remain in contact with their medical team and report new symptoms by telephone first (fever/cough/shortness of breath) [6].

As the situation changes, each center will be required to face unique ethical issues regarding allocation of resources, balancing the unknown risks of treatment versus benefits and measures taken to ensure the safety of patients, family members and staff. National and regional professional societies have a key role to play in disseminating best practice based on evidence as it emerges.

To conclude, COVID-19 pandemic represents a historical cancer care challenge for our oncology community. Preventive measures by the oncology staff, clinics, departments and patients themselves are necessary to decrease the number of infected cancer patients by COVID-19. General recommendations from local oncology societies with strategies for rapid communication of new data are mandatory to assure the best care for cancer patients during this COVID-19 pandemic.
